# Book Notices

**Published:** 1866-03

**Authors:** 


### EDITORIAL.

### BOOK NOTICES.

*The Physiology of Man: Designed to represent the existing state of Physiological Science as applied to the Functions of the Human Body.* By AUSTIN FLINT, Jr., M. D., Professor of Physiology and Microscopy in the Bellevue Hospital Medical College, etc. New York: D. Appleton & Co.

The first volume of this great work is before us. To be completed in four volumes, issued yearly, it will constitute the most exhaustive treatise on Physiology that has appeared in this country. Designed to rank beyond the student's text-book, it discards much of the didactic prolixity which encumbers many of the standard works; and presuming upon the anatomical knowledge of his readers, the author aims to present a clear statement of all that is known in relation to the Physiology of Man. The present volume treats of Proximate Principles; The Blood; Circulation; and Respiration. Without descending to all the minutiæ of experimental detail, the author has brought forward the results of the vast experience and knowledge acquired by vivisections performed on the lower animals. The chapters on Circulation and on Respiration are especially rich in the fruits of such observations. For the readers of the leading European medical journals, the statements of our author will possess very little novelty; but to the profession generally, the arrangement and authoritative statement of discoveries and facts which have been only recently and perhaps timidly announced, will be interesting. As the principal value of every new work on Physiology consists chiefly in the addition thus made to the previously existing stock of knowledge, we shall endeavor briefly to indicate some of the most important facts which are now for the first time placed in a tangible form before the professional public.

The chapter on Proximate Principles is more interesting than the corresponding chapter in the majority of physiological works. Under the head of *sugars*, the author discredits the theory that sugar and fat are oxidized in the lungs, or in the general system, for the purpose of keeping up the animal temperature. "The precise function of sugar and its mode of disappearance in the economy are not yet well understood. . . . We are only justified in saying that sugar is important in the process of development and nutrition, at all periods of life."

The question as to the possibility of the formation of fat in the system the author considers as definitely settled in the affirmative.

We are glad to see that the theory of Protein Compounds is rejected by Prof. Flint. He declares that "it is not a distinct chemical substance, for its composition is indefinite; nor a proximate principle, for it is produced artificially and by decomposition." The quantity of fibrin in the blood, "estimated by a process in which it is not exposed to dessication, is between 8 and 9 parts per 1000." He accepts Richardson's explanation of the cause of the fluidity of fibrin in the circulation, viz: "that the blood contains a small quantity of free ammonia, which has the power of maintaining the fibrin in its liquid condition." The whole subject is discussed at length in the chapter on the Blood, where are given all the arguments of different experimenters, *pro* and *con.* Prof. Flint, however, adopts the theory of Richardson as the only one fully substantiated by adequate observation. He denies the possibility that effused and coagulated fibrin is capable of organization. Effused fibrin may undergo a process of *fibrillization*, but this "is by no means an evidence of even commencing organization." Plastic lymph, which is capable of organization, is a substance quite different from fibrin.

The chapters on the blood are extremely interesting. The results of transfusion are related very fully, showing how wonderful is the vivifying power of this truly vital fluid. The following example is only one out of many: "Blood was passed from a living dog into the carotid of a dog just dead from peritonitis. The animal was so far revived as to sustain himself on his feet, wag his tail, etc., and *died a second time*, twelve and a half hours after. In this experiment insufflation was employed in addition to the transfusion." P. 99.

The author adopts Robin's explanation of the remarkable tendency of the corpuscles of fresh blood to arrange themselves in rows like rolls of coin, as observed with the microscope. "Shortly after removal from the vessels, there exudes from the corpuscles an adhesive substance which smears their surface and causes them to stick together. Of course the tendency is to adhere by their flat surfaces." P. 111.

The doctrine of the development of the red corpuscles from the colorless corpuscles meets no favor from Prof. Flint. "The red corpuscles appear before the leucocytes (white corpuscles) are formed. . . . It is most reasonable to consider that the red corpuscles are formed by a true *genesis* in the sanguineous blastema. . . . There is furthermore no sufficient evidence that any particular organ or organs have the function of producing the blood corpuscles. . . . Regarding them, as we certainly must, as organized bodies which are essential anatomical elements of the blood, it is difficult to imagine what reasons based on their function, should lead physiologists to seek so persistently after an organ for their destruction." (P. 120.) "The function of the leucocytes is not understood. The supposition that they break down and become nuclei for the development of red corpuscles, which at one time obtained, is a pure hypothesis, and has no basis in fact."

An entire chapter is devoted to a discussion of the causes of the coagulation of the blood. The experiments of Richardson and of his opponents are reviewed at great length, bringing the reader at last to the following conclusion: "When, as happens in the interior of the body, the blood coagulates under circumstances where the process will not admit of direct experimentation as far as the evolution of volatile substances is concerned, the best we can do is to apply, as far as possible, the facts which are proven with regard to coagulation out of the body, when the phenomena can be minutely studied. Here, at least in the human subject and in animals, it seems demonstrated to be due to the evolution of ammonia." P. 166.

The chapters on the Circulation present a full discussion of the phenomena attending the movement of the blood. The experiments of all the leading observers, from the days of Hippocrates to our own epoch, are passed in review, affording much that will interest those who have not access to the original authors. The Professor differs from Dalton with regard to the shortening and elongation of the heart. Dr. Dalton teaches that during the systole of the organ the ventricles are elongated. That the apex of the heart is protruded during the ventricular systole is true, but Flint has shown that this is due to the synchronous distention of the great vessels leading from the heart, and that during the systole the ventricles are actually shortened. This fact was ascertained by suddenly cutting out the heart of a warm-blooded animal, and pinning its base with two needles to a thin board. With every ventricular contraction the point of the organ was visibly retracted.

The recent experiments of Marey with the "cardiographe" are fully detailed. Our limits will not permit anything more than this allusion to this most ingenious and perfect method of registering the movements of the heart, making the organ write its own story by a process not unlike that by which the telegraphic indicator impresses upon paper an impulse transmitted from a distant station.

An interesting series of experiments is given to illustrate the influence of the nervous system upon the movements of the heart. The author seems to doubt the importance attributed by some to the influence of the cardiac plexus. As for the influence of the pneumogastric nerves, "they undoubtedly perform the important function of regulating the force and frequency of its pulsations." (P. 235.) In respect of sudden death from blows upon the epigastrium, he inclines to the belief "that in such accidents the symptoms are due to direct injury of the heart. An additional argument in favor of this view is founded on our knowledge of the mode of operation of the sympathetic system. The effects of stimulation or irritation of this system are not instantaneously manifested, as is the case in the cerebro-spinal system, but are developed slowly and gradually." P. 239.

With the exception of a recital of Marey's experiments upon the pulse, the chapter on the arterial circulation contains little that is new. For an account of Marey's sphygmograph we must refer our readers to the work itself; our limits will not permit us to transcribe the description of the delicate and complicated apparatus by which the pulse is made to trace on paper its actual force and form.

To the contractility of the smaller arterial branches our author ascribes the regulation of the capillary circulation. While refraining from a discussion of the phenomena of inflammation, he dissents from the opinion that this "modification of nutrition can be induced under our very eyes, simply by the application of irritants. With these views, microscopic researches on the *state of the blood and blood-vessels* in *inflammation* do not assume the importance which is attributed to them by many authors." P. 299.

Under the head of local peculiarities of the circulation, we notice that the Professor does not agree with those who consider any change in the quantity of blood within the cranial cavity "a physical impossibility." Among the organs provided with erectile tissue is now classed the uterus, since the experiments of Rouget, "who has lately shown by injections that the uterus is capable of erection like the penis." (P. 336.) "That the contractility of the lung tissue offers no impediment to the circulation is proved by the fact that an injection passes through the capillaries of the lungs as easily when they are collapsed as when they are inflated." P. 341.

While the section on Respiration contains much that is interesting, it presents little that is new. The subject of the diffusion of oxygen through the lungs and the removal of carbonic acid by respiration is not yet extricated from the difficulties that surround its comprehension. The concluding chapter on the relations of respiration to nutrition forms the most instructive part of this division of the work. Presenting a summary of the author's paper, originally published in the *Am. Journal*

*of the Medical Sciences*, Oct., 1861, it treats of the relations of oxygen and carbonic acid to nutrition; showing that oxygen, carried by the blood to the tissues, is by them consumed in some way that is not yet fully understood; while, in the words of Collard de Mortigny, "the carbonic acid expired is a product of assimilative decomposition, secreted in the capillaries and excreted by the lungs." From this view it naturally follows that the *besoin de respirer*, or *want of oxygen*, has its seat in the tissues rather than in the lungs or in the heart. This proposition is supported by the recital of a number of interesting experiments by vivisection, for which we must refer the reader to the pages of the work itself.

In conclusion, we cannot but congratulate the members of the medical profession upon the advancement of science which may be confidently expected as a result of the labors of such men as Prof. Flint. Though he may yet find it necessary to modify and to amend much that he has written, the evident zeal and candor with which his researches are conducted cannot fail to secure for his volumes a most cordial reception.

For sale by S. C. Griggs & Co., 41 Lake street.

---

*Chloroform and its Administration.* By ARTHUR ERNEST SANSOM, M. B., London. Philadelphia: Lindsay & Blakiston. 1866. Price, $2.25.

We cannot perhaps better give an idea of the merits of this book than to glean from it some of its teachings. It treats of the history of the discovery of anæsthetics, which shows that the boon has been sought by different persons through many years, and that the discovery has been long dawning upon the human mind, in accordance with what is perhaps the universal law, that great discoveries are not made suddenly, but are the consequences of generative antecedents, which render them possible, and from which they flow. As relates to the dangers of chloroform, statistics show that one death occurs in about 17,000 administrations. The author, however, thinks a considerable percentage of these die from fear or shock, and not from the effects of chloroform. In support of this view, he relates the following incidents: "In the first case, in which Dr. Simpson proposed to try the effects of chloroform in a surgical operation, a boy was to be cut for stone. Just as the preliminaries were arranged, the boy died. Not a breath of chloroform had been given. If it had, the birth of the anæsthetic would have been its death. In another case, an apparatus for administering chloroform was applied to a patient about to be operated on. Suddenly he died. All around thought chloroform had brought about the result, but it was found that the valve was closed and not a whiff of chloroform had entered the lungs. Such accidents were not wanting before chloroform was thought of. It is told of Desault, that just as he was once about to perform lithotomy, he traced with his finger a line on the skin of the patient—the man shrieked and fell dead. A similar result occurred when Chopart was about to perform a simple operation. Another occurred in the practice of Mr. Stanley." In all about three hundred deaths have been reported, and of these 44 per cent died before the operation had commenced; 34 per cent during its performance, and 20 per cent shortly after its completion. There seems to be some doubt whether the use of chloroform has lessened the ratio of mortality from surgical operations. In amputations for severe injuries there is no improvement in late years, and the author says, that "in severe accidents necessitating an immediate operation, I believe we should hesitate before administering chloroform." Surgical operations for disease are, however, much more successful than they formerly were, but this is unquestionably in good part due to the improved medical (and especially hygienic) treatment that such cases now receive. The chapter on the chemistry of chloroform is full of interest and instruction, and especially is this true in what relates to its adulterations and impurities, which, however, is too lengthy to transcribe, while it is difficult to abridge. In its administration, it is recommended that an inhaler that will graduate the proportions of chloroform and air should always be used. We think the author exaggerates the danger of inhaling it from a napkin. We have reason to believe that anæsthetic agents have always been administered almost exclusively in this way in this city, and we have yet to know of the first fatal result from their use; and though the experience of a single person is entitled to little weight in determining this question, even the negative testimony of a whole city during so long a period is worthy of consideration. The instrument is not so essential as that the person who administers the remedy should be possessed of knowledge and judgment. It has been more fatal to males than females—to those in middle life, than to the young or the aged—and more to the robust, than to the feeble and infirm. The diseases which increase the danger of its use are delirium tremens, fatty degeneration of the heart, uræmia and pyæmia, shock, hysteria and pulmonary disease, characterized by "general congestion and acute hyperæmia;" while, on the other hand, we are told that "patients suffering from consumption are, as a general rule, among the very best to submit to chloroform." The skin and tissues around the eye, rectum, genital organs and nails, longest retain their sensibility, and in the effort to overcome this, the use of the remedy is sometimes carried too far, which accounts for the relative frequency of fatal accidents from inhaling chloroform for trivial operations on these parts. The average amount inhaled in fatal cases in which it has been known, has been about one and one-half drachms—the smallest amount being but fifteen drops. To resuscitate from apparent death, artificial respiration, warmth and friction are relied on, while other stimulants are declared to be useless. The work concludes with chapters on anæsthetic mixtures, on the methods and practical rules for the administration of chloroform, and its uses in surgery, obstetrics and dentistry. It is a book that should be carefully read by every practitioner of medicine, surgery and dentistry. I.

For sale by S. C. Griggs & Co., 41 Lake street.

---

We have received from Dr. R. H. Ward, of Troy, N. Y., "The Transactions of the Medical Society of the State of New York for the Year 1865;" and from the author, Prof. J. C. Nott, of Mobile, Ala., a pamphlet, entitled "Contributions to Bone and Nerve Surgery."

*The Principles and Practice of Medicine.* By AUSTIN FLINT, M. D., Professor of the Principles and Practice of Medicine in the Bellevue Hospital Medical College. Philadelphia: Henry C. Lea.

This volume is for the most part made up from the lectures of Prof. Flint, and will be highly valued by the college graduates who have had the pleasure of listening to his instruction. The material is well classified, and is arranged very conveniently for reference. The work is chiefly valuable as a record of the experience and opinions of its author, and as such it is interesting. As contributing anything in advance of the teachings of other similar text-books, its value is very slight. Some of the topics are treated in a rather superficial manner, e. g., the chapter on Cholera, which is very unsatisfactory as compared with the illumination which might have been reflected upon the subject by proper reference to the physiological discoveries described in the work of the younger professor.

For sale by S. C. Griggs & Co., 41 Lake street.

---

We have received the following new medical journals: *The Savannah Journal of Medicine*, a monthly publication, edited by Drs. J. Harris, J. B. Read and J. G. Thomas. *The Richmond Medical Journal*, edited by Drs. E. S. Gaillard and W. S. McChesney, a monthly journal, published in Richmond, Va. *The Medical Reporter*, a semi-monthly record of Medicine and Surgery, edited by Drs. J. S. B. Alleyne and O. F. Potter, of St. Louis.

